# The nurse coordinator role: fulfillment of the nursing profession's compact with society

**DOI:** 10.1186/s13584-018-0280-6

**Published:** 2019-01-04

**Authors:** Anne H. Gross, Jessica Driscoll, Laura Ma

**Affiliations:** Dana-Farber Cancer Institute, 450 Brookline Ave, Boston, MA 02215 USA

**Keywords:** Nurse coordinator, Nurse navigator

## Abstract

The implementation of a new role in healthcare teams frequently emanates from emerging or changing needs in the care delivery system or expressed needs of clinicians, patients or caregivers. In this commentary on the experience of the nurse coordinator role in Israel we suggest based on similar experiences in the United States, that effective implementation is accomplished when the functions of the role are well delineated with respect to other members of the team and informed by the needs of patients, their caregivers and clinicians. The outcomes expected from those performing the role should be established and measured over time.

## Main text

Using a phenomenological approach, Monas and her colleagues describe the experiences and views of nurse coordinators’ performance in the Sharett Oncology Institute of the Hadassah Medical Center at Ein Kerem in Jerusalem. They assert that despite the fact the role is considered an essential component of the oncology care team in Israel, a paucity of literature exists on the subject [1]. The authors’ aim was to examine how the nurse coordinator role is implemented in various oncology settings in the hospital, describe the actual performance of the nurses and document study participants’ perceptions of the role as carried out in their tertiary hospital in Israel. By examining the nurse coordinator role, the intent was to deepen the current understanding and put forth recommendations for clearer role definition.

In this commentary, we offer a historical frame of reference and thoughts on approaching the challenges of developing and implementing a new role like the one reported in this descriptive study. We comment on study design and methods and share experiences from the United States in articulating the practice scope, competencies and skills of nurses who coordinate, navigate and facilitate oncology care across clinical teams, across the healthcare system and with patients and caregivers. The challenges illuminated by this study, have been similarly experienced in oncology settings in the US and across the US healthcare system.

### Historical frame of reference

In her early writings, Florence Nightingale, the founder of modern nursing, argued that professions belong to the societies within which they develop, and it is those societies that determine the professional skills and knowledge that are needed [2]. In other words, as a profession, nurses must continuously advance and adapt to meet the needs of the societies or patients they serve. The American Nurses Association (ANA) promulgated a Social Policy Statement (1980, 2003) to articulate the covenant nursing has with society. Incorporating Nightingale’s thinking and that of twentieth century nurse theorists, the ANA Social Policy Statement describes the profession as dynamic, and relevant only to the extent that it evolves in concert with the changing needs of society [3]. Throughout our history, nurses have repeatedly expanded knowledge and adapted practice to meet the evolving needs of patients and the health care system. Through those efforts the roles of bone marrow transplant nurses, critical care nurses, hospice nurses, and others have emerged. Therefore, it is no surprise that the oncology nurse coordinator or nurse navigator role has recently emerged in our societies to address the changing needs of patients, caregivers and clinical teams in an increasingly complex system, with complicated treatment regimens across the domains of surgery, medical oncology palliative care and radiation oncology. As a relatively new nursing role, the authors note, and we concur, that explicit role definition and competencies associated with the role must be defined.

## Study design, methods and outcomes

The phenomenological study to explore participant experiences and views of the nurse coordinator role included participants from one cancer hospital in Israel (nurses, nurse coordinators and physicians), nurses from the Ministry of Health and the Israel Cancer Association and members of the Israel Organization of Oncology Nurses. Patients and caregivers were not included in the study, even though their experience of care and ability to navigate the health care system effectively are the primary focus of the nurse coordinator role. Our experience is that patients’ firsthand perspectives are frequently different and more illuminating than clinicians’ untested assumptions about their needs. Therefore, if the intent of the study was to better understand what nurse coordinators do at Sharett Oncology Institute, and strengthen the role based on patient and clinician needs, the direct input of patients and caregivers would have added important perspective to the results.

Given the questions posed by the study authors, content analysis rather than the phenomenological approach may have yielded a more structured identification and categorization of the nurse coordinator functions from the participants. This in turn may have been more directional to the team in answering the study questions. That said, through the exploration of participants’ experiences, the study did validate the importance of the role, illuminated challenges experienced by nurse coordinators and others and named the tensions that have arisen with other nurses in caring for the same patients.

Interestingly, reports of similar role implementations in other countries, also tend not to be informed by patient input. An integrative review of eighteen studies reporting outcomes of nurse navigator roles in the US, Canada and Sweden describes some attempts at soliciting patient satisfaction with the role, once in place, but it was not consistent across all studies and the patient feedback was not gathered prior to implementing the roles [4]. An important contribution to the literature and to the continuing evolution of this role would be an examination of patients’ and caregivers’ views of their care coordination and navigation needs to be certain that the roles we embed in the clinical team meet their articulated needs.

### The nurse coordinator role (Israel) and the nurse navigator role (United States)

Monas and her colleagues trace the origins of the nurse coordinator role in Israel (1976) and specifically at the Sharett Oncology Institute (late 1990’s). The emergence of a similar role in the US, titled nurse navigator, also occurred in the early 1990’s in the specialty of oncology. Educated in a holistic approach to patient care, nurses have been strong advocates for patients and have fulfilled care coordinating functions through time. However, as new, high risk, complex oncology treatments were being translated to the bedside and healthcare systems around the world were becoming more and more complex, a specific need emerged. A clinician with a singular focus on supporting teams by coordinating care across the system and assisting patients through treatment by removing barriers, addressing gaps, educating and assisting them to navigate the system has become essential. Oncology nurses, with their holistic approach to patient care are the obvious discipline to meet this need and the role has taken root in Israel, the US and other parts of the world.

Challenges with implementing the nurse coordinator role described in the study included a lack of standardization, feelings of isolation from the care team amongst nurses in the role, tension between clinical nurses and the nurse coordinators emanating from role overlap and confusion and limited organizational support. Many of these same challenges have been experienced in the US and specifically in our cancer center. An internet search of terms yields numerous titles associated with the core functions of the nurse navigator/coordinator role. Their implementations have specific emphases corresponding to the practice setting and populations served. However, there are overarching components and competencies of the role that are similar across specialties in oncology, and countries around the world. The study outcomes associated with the nurse navigator/coordinator role in the US, Canada and Sweden were in the areas of patient advocacy and case management, improvements in time from diagnosis to treatment, decrease in patient anxiety and possible, though not quantified, reduction in health care costs.

In 2012, the US Oncology Nursing Society (ONS) and the Oncology Nursing Certification Corporation (ONCC) conducted an oncology nurse navigator role delineation study to increase the understanding of the role by defining the tasks, knowledge areas and skills of nurses in the role. Three hundred and thirty nurse navigators participated. The overlap of skills and knowledge between the nurse navigators and general oncology nurses was identified in that study [5] and the Oncology Certified Nurse (OCN®) was the recommended certification. ONS and ONCC published another role delineation study in 2016 examining changes in the navigation role since the 2012 report. In 2017 they published the first core competencies of the oncology nurse navigator role [6] and the ONCC is currently evaluating a certification option to acknowledge the unique expertise of nurses in this role. To date, the measurable, sustained impact of nurse navigators’ interventions on the patient experience, the clinical team experience, healthcare costs and quality has not been fully documented in the literature and is the focus of ongoing study in the US.

## Conclusions

The implementations of the oncology nurse coordinator and oncology nurse navigator in Israel and the US have taken similar paths and raised similar challenges, however the importance of the role has been documented in both countries. If it is correct to assume the Sharett Oncology Institute of the Hadassah Medical Center experience is representative of other Israeli institutions, a logical next step would be to systematically define the process and outcome measures of success expected from those who work as nurse coordinators in Israel, based on patient, caregiver and clinical team input. From there the scope of the nurse coordinator role, the specific competencies required and the connectivity to other nursing roles in the healthcare system could be better delineated. The US may be a little further along in this process, but not by a significant measure. Continued efforts to clarify the role functions and differentiate nurse coordinators and navigators from other nursing roles would be a great service to the nursing profession, to the healthcare teams within which we work, and ultimately to the patients and caregivers who rely on so heavily on our services. And finally, continued dissemination of these efforts in the literature and at conferences will be invaluable to others around the world who strive to effectively embed the functions of a nurse coordinator in their own clinical settings.
